# A Comparison of Long-Term Care Use by Community-Dwelling Older Adults With and Without Dementia

**DOI:** 10.1093/geroni/igab046.1348

**Published:** 2021-12-17

**Authors:** Yuchi Young, Arianna Stone, Kuo-Piao Chung, Hsiu-Hsi Chen, Yen-Po Yeh

**Affiliations:** 1 State University of New York at Albany, Rensselaer, New York, United States; 2 University at Albany School of Public Health , Albany , New York, United States; 3 National Taiwan University, School of Public Health, Taipei, Taipei, Taiwan (Republic of China); 4 National Taiwan University, National Taiwan University, Taipei, Taiwan (Republic of China); 5 Changhua County Public Health Bureau, Changhua City, Changhua, Taiwan (Republic of China)

## Abstract

Introduction. This study compares long-term care (LTC) use among community-dwelling older adults with and without dementia. Methods. Participants (n=14,483) were aged 65+ residents of Changhua County, Taiwan who qualified for LTC services. Data were collected (4/1/2017-10/26/2018) through health assessments. Multivariate logistic regression quantifies the study aim. Results. Preliminary results show that on average participants with dementia are older than people without dementia (81.1 vs. 80.5; p<.001), more females (13.4% vs. 8.0%; p<.001), higher mean ADL (12,4 vs. 9.8; p< .001) and IADL (21.4 vs. 17.8; p<.001), and lower mean comorbidity (2.5 vs. 2.8; p<.001). Multivariate regression results indicate people with dementia use twice the health-related LTC services than their counterpart (OR= 2.0; 95% CI 1.90–2.14). Discussion. People with dementia use more health-related LTC services. Future dementia studies should examine the pattern of non-health-related LTC services concomitant with health-related services, so that person-centered care can be tailored to foster aging-in-community.

